# Sequence squeeze: an open contest for sequence compression

**DOI:** 10.1186/2047-217X-2-5

**Published:** 2013-04-18

**Authors:** Richard CG Holland, Nick Lynch

**Affiliations:** 1 Eagle Genomics Ltd, Babraham Research Campus, Cambridge, CB22 3AT, UK; 2 AstraZeneca UK, Alderley Park, Macclesfield, SK10 3QX, UK; 3 Pistoia Alliance Inc, 401 Edgewater Pl., Ste. 600, Wakefield, MA, 01880, USA

**Keywords:** Next-generation sequencing, Cloud computing, Data compression, Open innovation, Big data, Pre-competitive, Competition

## Abstract

Next-generation sequencing machines produce large quantities of data which are becoming increasingly difficult to move between collaborating organisations or even store within a single organisation. Compressing the data to assist with this is vital, but existing techniques do not perform as well as might be expected. The need for a new compression technique was identified by the Pistoia Alliance who commissioned an open innovation contest to find one. The dynamic and interactive nature of the contest led to some novel algorithms and a high level of competition between participants.

## Background

In October 2011 the Pistoia Alliance [1] announced a contest to source a new compression technique for the management of data generated by next-generation sequencing machines. The volume of sequencing data produced is growing rapidly [2] and is putting pressure on existing techniques for storage and data transfer. New techniques are available which use reference-based compression to significantly improve ratios, such as the European Bioinformatics Institute’s CRAM algorithm [3], but all such algorithms available at the time of the contest were lossy (i.e., they discard information that the algorithm considers unimportant). The Pistoia Alliance was concerned with finding a technique that was lossless – that is, able to exactly reproduce the input data upon decompression without error or omission. This desire for high quality compression was driven by the demands for distributed science and the needs to send data files from the sequencing team to a remote analysis team.

A primary goal of the contest was to ensure that the wider community would benefit from the discoveries made. All entries were required to be submitted under an open-source licence which would permit unrestricted use by anyone regardless of whether they worked for commercial or non-profit organisations. At the end of the contest the source code for all entries was made available via links from the contest website [4].

The contest featured a dynamic leaderboard on its webpage which used cloud computing technology to automatically assess and score every entry in real time as soon as it was submitted. When each assessment was complete, an email was sent to the entrant and the leaderboard updated to illustrate their performance.

## Main text

The automated judging mechanism behind the dynamic leaderboard was the key technical feature of the contest. Entrants were asked to submit their compression and decompression code in an Amazon Web Service (AWS) S3 bucket whose contents conformed to the format specified on the contest website. The bucket had to contain all dependencies and external data that the entry required.

Submission was via a web-based form which noted the entrant’s details and a reference to their entry’s location in AWS S3. A single AWS instance was kept running to monitor the database for new entries at five-minute intervals. When a new entry was detected, a new AWS instance would be started to judge it. The judging instances were discarded after each use in order to minimize the risk of cross-contamination between judging cycles. The single-use approach also allowed multiple entries to be judged in parallel.

Each judging instance contained a simple script which controlled the judging process. It operated as follows:

1. Download the entry

2. Set up a the contest data (a random extract from the 1000 Genomes Project [5])

3. Secure the firewall

4. Run the entry in compression mode

5. Measure CPU and memory usage

6. Assess the compression ratio

7. Run the entry in decompression mode

8. Check that the total combined output files contain exactly the same information (header, sequence, and quality lines) as the input files

9. Update the results database

10. Email the results

## Discussion

The real-time judging and dynamic leaderboard had a clear motivational effect on entrants as they were able to see immediately how their entries compared with their peers. In many cases this led to entrants submitting multiple entries as they attempted to regain pole position; thus encouraging further innovation and development of their ideas in a bid to stay ahead of the competition. A veritable flurry of activity occurred in the closing week of the contest where the most enthusiastic entrants were submitting up to three new attempts each per day. Interestingly by comparison, none of the entrants who waited until the last minute to submit their single attempts ended up further than halfway up the final leaderboard.

Entries were ranked in a number of categories without an overall score. The aim of the contest was not to create a solution that came top of any one category, but to create one that performed well all-round. This required the participation of a human judging panel in order to assess, in their professional opinion, which entry had contributed most to progress in the field, as well as looking at the source code and concept to predict suitability for production-scale deployment.

The overall winner of the contest was announced in April 2012 and a selection of entries are shown in Table 1. James Bonfield (Wellcome Trust Sanger Institute, UK) produced a technique [6] which relied on the compression of BAM alignment files rather than the original FASTQ data. The use of FASTQ had been mandated at the outset of the contest to remove any problems in comparing performance between vastly differing input formats. To obtain the BAM files, Bonfield first aligned the FASTQ against a reference human genome that had been bundled with his entry. This semi-reference-based approach led to good overall performance in most of the contest’s categories – memory usage, speed, and ratio – whilst maintaining total data integrity without any round-trip loss. It was notable that so many entrants achieved full lossless compression that all those that did not could be safely removed from the running at the start of the final judging process without negatively impacting on the remaining pool of ideas.

**Table 1:** A selection of entries vs baseline algorithms

**Entry number and entrant**	**Best in category**	**Result**	***bzip2 *result**
101: James Bonfield	Compression ratio	0.1141	0.3007
61: James Bonfield	Compression time	109.9	1020.97
28: Ryan Braganza	Compression memory	15040	5200
7: James Bonfield	Decompression time	100.91	104.5
28: Ryan Braganza	Decompression memory	13472	5008

The results from running *bzip2* are shown against the winning entries in each category of the contest. Full results from all entries, including links to their source code, are available on the Sequence Squeeze website [4]. Compression ratios are the ratio of compressed file size to original file size (smaller is better). Times are in clockface seconds. Memory usage is peak in kilobytes. Entries with less than 100% round-trip accuracy are excluded.

The reference-based approach was not mandated by the contest, but was a common feature amongst high-ranking entrants including Bonfield and Matt Mahoney (Dell Inc.) [7]. However, their techniques do not work at all in the absence of a reference genome. Reference-based approaches were not actively promoted because the organizers originally wished to see a solution that would work regardless of the source of the sequence. In the end, entries compressing the sequence data in isolation did not fare so well. The baseline entries using *gzip* and *bzip2* achieved a consistently high placement in all categories.

The organizers never revealed the exact format of the test data header lines (the only customizable part of the FASTQ specification) and thus no entries would have been over-tuned to just one format. This helped make the entries portable and robust when faced with unexpected header line formats.

Bonfield did not actually have one winning entry; rather he had a set of related entries that populated most of the top positions in each category of the contest. This reflected a key outcome, that a one-size-fits-all approach is simply not appropriate in the compression of sequence data. Some organisations may need faster compression times (for quick storage of large volumes), some might want faster decompression (for later review of the data), whereas others might need better compression ratios (for regular network transfer). The contest demonstrated that none of the algorithms would be able to deliver on all fronts –variations or configurations could improve performance in one single category, but never more.

## Conclusion

The contest attracted in excess of 100 entries, but from a field of less than 20 entrants. The leaderboard clearly encouraged entrants to make repeated attempts to innovate and climb above their peers in the table of results. Using contests to drive innovation has been done before (e.g., Assemblathon [8]), but the dynamic leaderboard feature of Sequence Squeeze is clearly very useful as it gives transparency and immediacy to a competitive process which could otherwise be opaque and secretive. However, in the case of Sequence Squeeze, the lack of clarity on objective criteria for the overall winner, as opposed to subjective opinion of the judges, is an area that would need to be addressed.

The end result of the contest was a set of brand new compression algorithms for next-generation sequencing data, all of which are fully open-source and available for the community to use and build upon with their own ideas. This open-source requirement laid down by the Pistoia Alliance ensured that the whole community would benefit from the open innovation that it was promoting via the contest, and the data compression lessons learnt in the process could be shared with everyone.

## Abbreviations

AWS: Amazon Web Services; S3: Simple storage service.

## Competing interests

The authors declare that they have no competing interests.

## Authors’ contributions

RH created the automated judging environment, promoted the contest, managed the entries and the judging process, and drafted the manuscript. NL led the contest initiative at the Pistoia Alliance and reviewed the manuscript before submission. Both authors read and approved the final manuscript.
